# Supplementary material for: A Thai Traditional Triple-Fruit Formulation “Phikud Tri-Phon” May Provide Fat Loss and Nutritional Benefits
Source: Foods. 2022 Oct 2;11(19):3067. doi: 10.3390/foods11193067 (PMC9563312; doi:10.3390/foods11193067)
Supplement: Supplementary file 1 [file foods-11-03067-s001.zip › foods-1899094-supplementary.pdf]

Supplementary data

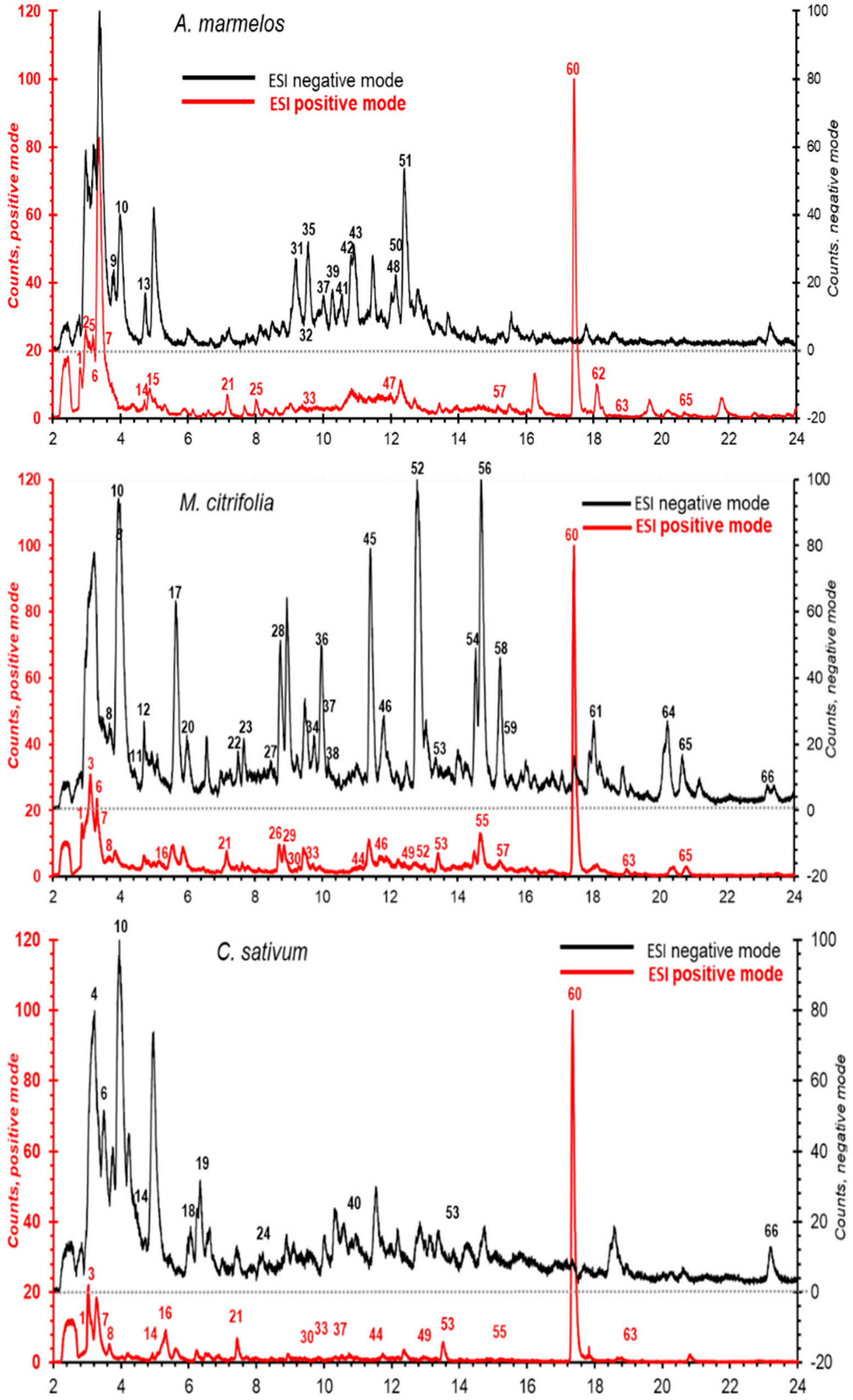

Figure S1. Total ion chromatogram of 10 mg/mL aqueous extract of *A. marmelos* (A), *M. citrifolia* (M), *and C. sativum* (C) by LC/MS operated in ESI negative mode and in ESI positive mode. The characteristics of each peak are in Table 3.
